# Administration of fibrinogen concentrate for refractory bleeding in massively transfused, non-trauma patients with coagulopathy: a retrospective study with comparator group

**DOI:** 10.1186/1471-2253-14-109

**Published:** 2014-11-26

**Authors:** Santiago R Leal-Noval, Manuel Casado, Victoria Arellano-Orden, Reginald Dusseck, Javier Bautista-Paloma, Manuel Muñoz, José Naranjo-Izorieta, Antonio Puppo Moreno, Aurelio Cayuela

**Affiliations:** Critical Care Division, Hospital Universitario “Virgen del Rocío” and Instituto de Biomedicina IBIS, Avenida Manuel Siurot s/n, 41013 Seville, Spain; Pharmacy Division, Hospital Universitario “Virgen del Rocío” and Instituto de Biomedicina IBIS, Avenida Manuel Siurot s/n, 41013 Seville, Spain; Transfusion Medicine, University of Málaga, Málaga, Spain; Statistics and Design Division, Hospital del Valme, Seville, Spain

**Keywords:** Anaemia, Bleeding, Clauss method, Fibrinogen concentrate, Goal directed therapy, Massive transfusion protocol, Thromboelastometry, ROTEM, Thromboelastography, TEG, FIBTEM, Transfusion

## Abstract

**Background:**

This retrospective, single centre study was conducted to investigate the efficacy of fibrinogen concentrate (FBNc) in decreasing blood requirements and reaching optimal fibrinogen level, in non-trauma, massively transfused, bleeding patients with coagulopathy.

**Methods:**

Over a 3-years period, all patients for whom a massive transfusion protocol was activated and had received ≥4 units of allogeneic blood components within a ≤4 h period, were included. Patients were classified according to whether they received FBNc or achieved an optimal fibrinogen level of ≥2 g/L within 24 h after FBNc administration.

**Results:**

Seventy-one patients received 2 [2,4] g of FBNc (FBNc group) and 72 did not (comparator group). FBNc was administered after transfusing 5 [5,9] blood component units, 3 [2,6] hours after massive transfusion protocol activation. Linear regression analysis showed that SOFA (AOR 0.75 [95% CI:0.08-1.43]) and admission fibrinogen level (AOR -2.7 [95% CI:-4.68 – -0.78]), but not FBNc administration, were independently associated with total transfused units. There was a significant inverse relation between both admission and target fibrinogen levels, and total transfused components. Logistic regression showed a direct relationship between admission fibrinogen level and achieving a target level ≥2 g/L (AOR 3.29 [95% CI;1.95-5.56]). No thromboembolic events associated with FBNc were observed.

**Conclusions:**

In massively transfused, non-trauma patients with coagulopathy and refractory bleeding, late administration of low FBNc dosage was not associated with decreased blood transfusion or increased post-infusion fibrinogen level. Given that both fibrinogen upon admission and target fibrinogen levels were associated with decreased blood transfusion, earlier administration and higher doses of FBNc could be needed.

## Background

Fibrinogen is the most abundant coagulation factor and the first one in reaching critical low levels during severe bleeding [1]. In patients with major bleeding, requirements for fibrinogen are larger than for any other haemostatic protein [2]. Trauma and surgical bleeding patients often present low levels of fibrinogen, and bleeding volume and hypofibrinogenemia appears to be associated with poor clinical outcome [1, 3].

Replacement of acquired fibrinogen deficiency with fibrinogen concentrate (FBNc) in patients with massive haemorrhage seems to be more efficacious than plasma in decreasing bleeding and transfusion rate [3]. Additionally, the administration FBNc (20 mg/mL) offers the theoretical benefits of infusing up to tenfold more fibrinogen than fresh frozen plasma (FFP, 2–3 mg/mL), in less volume and time. However, plasma contains all clotting factors and most guidelines still recommend its administration [1, 4].

Supplementation with fibrinogen may be more effective when used as a part of an early goal-directed therapy in bleeding patients [5, 6]. In these cases, viscoelastic test-guided, early FBNc administration, avoiding unacceptable standard laboratory test delays, has been shown to decrease blood transfusion requirements [5–9] and to be cost - effective [8, 9].

Guidelines recommend plasma and/or FBNc administration for acquired hypofibrinogenemia in patients with severe bleeding and coagulopathy following surgery or major trauma [10–13]. However, its use as adjuvant therapy for patients requiring massive transfusion is not yet a widely approved indication for FBNc [7], even though many countries have licensed FBNc for treatment of congenital and acquired fibrinogen deficiencies [14].

The efficacy of FBNc in decreasing blood requirements in non-trauma clinical settings have been addressed in few studies, most of them without a comparator group [3]. Therefore, the evidence regarding indications, dosing, timing, efficacy and safety of FBNc administration in massively transfused non-trauma patients is scarce [15]. Nevertheless, the European guidelines for the management of severe perioperative bleeding recommends treatment with FBNc if significant bleeding is accompanied by at least suspected low fibrinogen concentrations or function (1C) [10]. A fibrinogen concentration <1.5–2.0 g/L or thromboelastrometry (ROTEM) / thromboelastrography (TEG) signs of functional fibrinogen deficiency should be triggers for fibrinogen substitution (1C) [10].

**Figure 1:**
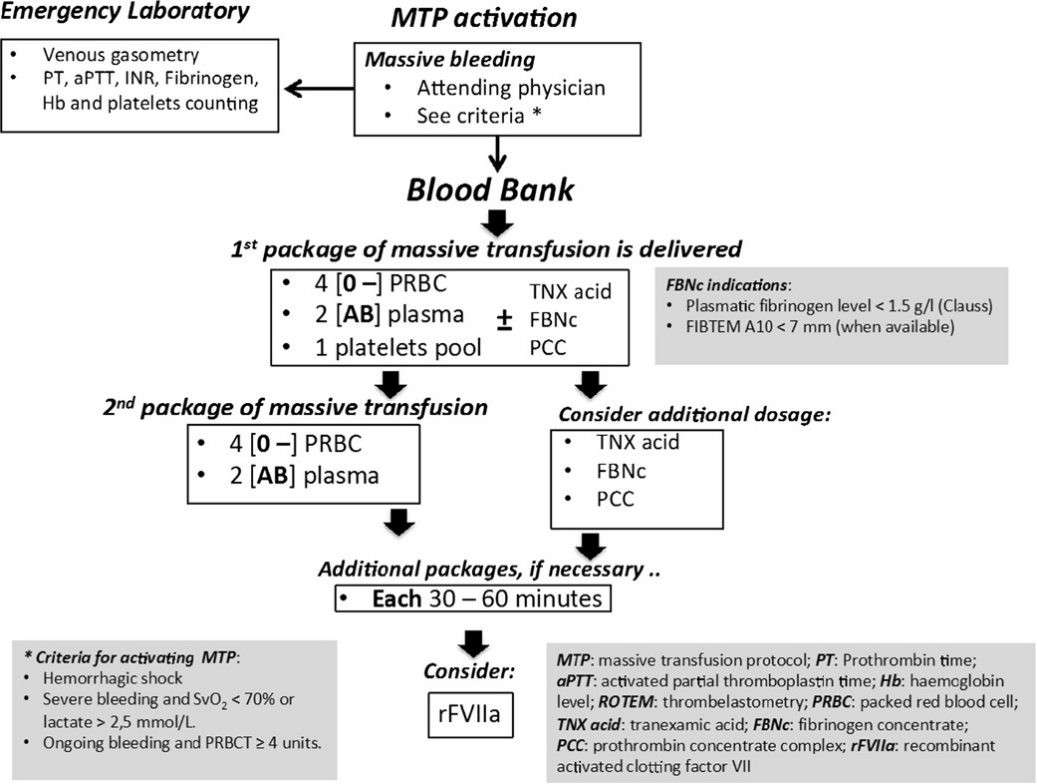
**Current massive transfusion protocol (MTP) for management of patients with massive haemorrhage at University Hospital Virgen del Rocio, Seville (Spain).**

Three years ago, our institution approved the use of FBNc, as a part of the massive transfusion protocol (MTP), after administration of the first transfusion package, in bleeding patients with fibrinogen levels of less than 1.5 g/L (Clauss method) (Figure 1). This retrospective, single-centre study with a comparator group assessed whether FBNc administration to massively transfused non-trauma patients with on-going bleeding could attain the target fibrinogen levels recommended by guidelines and reduce transfusion requirements (primary endpoints).

## Methods

### Setting and study design

This is a retrospective, single-centre, cohort study of patients admitted to the teaching hospital “Virgen del Rocío”, Seville, Spain, over a 3-years period (January 2011 through December 2013). The study was approved by the Hospital “Virgen del Rocío” Ethics Committee who waived the need for obtaining patients’ written consent.

The hospital performs complex procedures including cardiac, liver transplantation and gastrointestinal surgeries and has a transfusion guideline elaborated for the Transfusion Committee available on its intranet page. This guideline includes a massive transfusion protocol (MTP), which is activated by the patient’s attending physician (anaesthesiologist, intensivist, surgeon or emergency physician) (Figure 1). Blood components are indicated with restrictive criteria and guided by patient’s signs and symptoms, rather than merely by laboratory values. Alternatives to blood transfusion, including the administration of coagulation factors, are used according to the recommendations of the “Spanish consensus statement on alternatives to allogeneic blood transfusion: the 2013 update of the Seville Document” [12].

Massive bleeding was defined as the need for transfusion of more than 4 blood component units within a 4-hour period or bleeding leading to haemorrhagic shock, hyperlactacidemia and vena cava oxygen-haemoglobin desaturation.

Fibrinogen concentrate (FBNc: Haemocomplettan^**®**^, Riastap^**®**^; CSL Behring GmbH, Marburg, Germany) was only considered if persisting diffuse bleeding after the first massive transfusion package (4 packed red blood cell [PRBC] units, 2 fresh frozen plasma [FFP] units and 1 platelet [PLT] pool; (Figure 1), and hypofibrinogenemia was suspected or definitely detected by conventional laboratory tests (<1.5 g/dL by Clauss method or derived fibrinogen assay). Usually, 25–50 mg/kg of FBNc was administered in order to reach fibrinogen levels of 1.5 - 2.0 g/L, but never as a first-line therapy.

**Figure 2:**
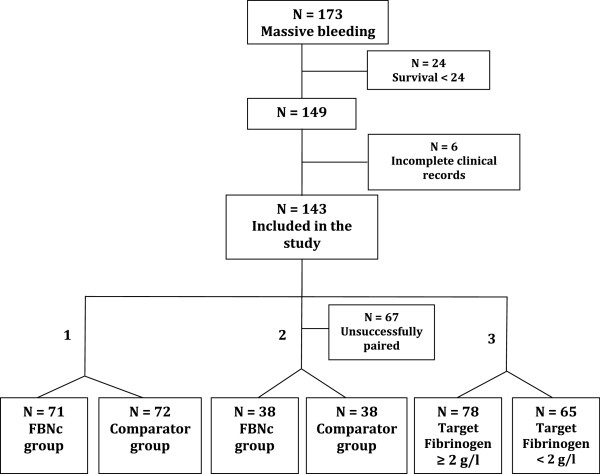
**Patients (N) included into a massive transfusion protocol.** Comparisons were performed between: (1) patients receiving (FBNc group) or not (comparator group) fibrinogen concentrate (FBNc); (2) patients receiving (FBNc group) or not (comparator group) FBNc and successfully pair-matched by SOFA, age and diagnosis; and (3) patients achieving or not a fibrinogen level ≥2 g/l

For the proposal of this study, all the patients who received 4 or more units of blood components over a 4-hours period during the peri-operative period or after admission to the emergency room or ICU were initially included. Exclusion criteria comprised patients who died within 24-hour from haemorrhage onset, because of uncontrolled multiple factors which led to early death could explain the primary outcome (flow chart, Figure 2), and those on oral anticoagulant therapy or with incomplete clinical records.

### Groups and variables

For each patient, a set of demographic and clinical variables including age, gender, severity of illness at the onset of bleeding (as assessed by SOFA: sepsis organ failure assessment), admitting diagnosis, length of hospital stay, complications, total number of transfused units (PRBC, FFP and PLT pools), and crude in-hospital mortality were retrospectively gathered. Multiple laboratory data including clotting tests and fibrinogen levels were also recorded. Both the total number of transfused units and laboratory tests were documented from 12 hours before to 12 hours after FBNc administration.

For clarity in data presentation, four diagnostic groups were considered: cardiac surgery, liver transplantation, gastrointestinal bleeding, and mixed group. Haemorrhagic episodes occurring at the operating room or ICU *vs.* general medical or surgical wards or emergency department were also differentiated.

The efficacy of FBNc administration was assessed by its ability at decreasing blood transfusion, as well as at attaining a target fibrinogen level of at least 2 g/L (Primary endpoints).

For the first primary endpoint, patients were classified according to whether or not they had received FBNc. Subjects with massive bleeding who did not receive FBNc formed the comparator group. Patients treated with FBNc accounted for FBNc group (Figure 2, bullet 1). The primary endpoint was the total number of allogeneic blood components administered since the MTP activation.

Additionally, given that severity of illness, diagnostic group and age may remain as strong confounders for determining the primary end point despite multivariate analysis, data were re-analysed after performing a matching process (Figure 2, bullet 2). Each patient from the FBNc group was pair-matched with other one from the comparator group, upon fulfilment the matching criteria of belong to the same diagnostic group, and present with the same SOFA score (±2) and age (±5 years).

Afterwards, for the second primary endpoint, we investigated those factors associated with reaching a target fibrinogen level of at least 2.0 g/L within a time period of 24 hours, regardless of whether they received or not FBNc. (Figure 2, bullet 3).

Secondary endpoints included the number of units of each individual blood component given (PRBC, FFP, and PLT pool), the length of hospital stay (days), and the rates of thromboembolic adverse events and crude in-hospital mortality. Medical records were carefully reviewed in order to detect any myocardial infarction, cerebral stroke, pulmonary thromboembolism and/or deep venous thrombosis occurring from MTP activation to hospital discharge.

### Statistical analysis

To detect a reduction of transfusion requirements of at least 2 ± 2 units after FBNc administration, with an 80% power (β-error) and a 95% confidence interval (α-error) for the nonparametric Wilcoxon rank sum test, data from at least 60 patients per arm (comparator and FBNc) would be needed.

Because most variables were non-normally distributed, data are reported as median (interquartile range [IQR]) and percentages. For continuous variables, comparisons between groups were performed with the nonparametric Kruskall-Wallis and Mann–Whitney tests, whereas Pearson’s chi squared test was used for the comparison of proportions. Continuous variables at different time periods (before and after FBNc administration, within-group comparisons) were compared with the nonparametric Wilcoxon rank sum test.

A general regression linear model (ANOVA), which provides both a regression and variance analyses for a dependent continuous variable, was developed for investigating variables independently associated with overall transfusion requirements. Factors associated with achieving fibrinogen level ≥2 g/L, within a 24-hours period after MTP activation, were investigated using a step forward logistic regression analysis.

All statistical analyses were performed using a computer software package with license (SPSS 18, SPSS, Inc., Chicago, IL), and a *p* value of less than 0.05 was considered significant.

## Results

Between 2011 and 2013 a total of 143 patients (60 [48, 69]) years old, SOFA 6 [4, 9], 76.6 % male) were treated for massive haemorrhage in our centre (72 received exclusively blood components, and 71 received blood components plus FBNc). Patients’ demographics and clinical characteristics are summarized in Table 1.

Overall, 134 out of 141 patients (95%) received the first package of blood components within 40 minutes from the activation of MTP. In contrast, the average time for administration of FBNc was longer and variable (3 [2,6] hours), because our MTP dictates that FBNc is to be administered always after transfusing the first massive transfusion package (Figure 1).

**Figure 3:**
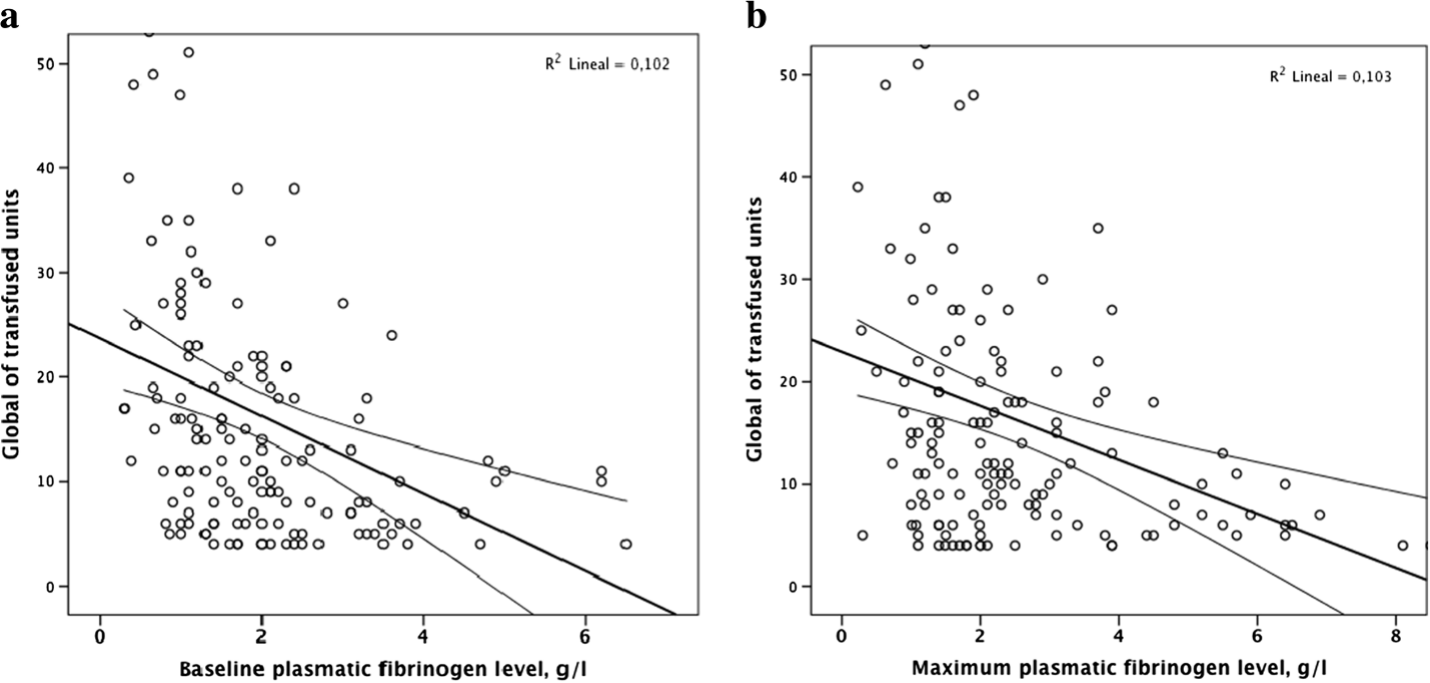
**Relationship between the global number of transfused units and fibrinogen levels upon admission (baseline) (a) and highest levels within 24-hours after activating massive transfusion protocol (b).**

There was an inverse and independent correlation between transfusion requirements and fibrinogen levels upon admission, as well as between transfusion requirements and maximal fibrinogen levels within 24-hours after activating MTP (Figure 3). An increase by 3 units of blood components transfused per each g/l decrease in fibrinogen upon admission was observed.

**Table 1:** **Characteristics of both cohorts of patients, receiving (FBNc group) or not (comparator group) FBNc**

Variables	FBNc N = 71	Comparator N = 72	P value
Age (years)	57 [44, 66]	63 [53, 72]	0.02
SOFA	7 [4, 10]	5 [3, 8]	0.03
Gender (males)	46 (65)	55 (76)	NS
Haemorrhage occurring outside the OR or ICU	25 (35.2)	24 (33.3)	NS
**Diagnoses**			
Cardiac Surgery	37 (51.4)	35 (49.3)	NS
Liver Transplantation	11 (15.3)	11 (15.5)	
Gastrointestinal Bleeding	16 (22.2)	15 (21.1)	
Other	8 (11.1)	10 (14.1)	
***Variables at beginning of the bleeding***			
pH	7.30 [7.29, 7.32]	7.31 [7.26, 7.3]	NS
Base excess (mEq/L)	-4.3 [-7.3, -3.0]	-4.0 [-6.2, 0.3]	0.04
Creatinine (mg/dl)	0.9 [0.7, 1.3]	0.9 [0.7, 1.6]	NS
Leukocytes (x 10^9^/L)	7.4 [4.7, 12.9]	10.3 [8.1,14.6]	0.04
Platelets (x 10^9^/L)	93 [49, 150]	100 [63, 144]	NS
Haemoglobin (g/L)	85.0 [71.5, 103.0]	70.0 [64.0, 75.0]	0.001
Prothrombin time (s)	19.2 [14.1, 31.6]	16.7 [14.7, 20.3]	NS
Thromboplastin time (s)	46.1 [34.1, 97.3]	43.5 [34.0, 55.5]	NS
Fibrinogen (g/L)	1.3 [0.9, 2.2]	2.0 [1.4, 3.1]	0.001
INR	1.7 [1.2, 2.8]	1.4 [1.2, 1.7]	0.01
***Outcome variables***			
In-hospital mortality	29 (40.8)	20 (27.8)	NS
Length of hospital stay (days)*	14 [7, 30]	23 [17, 33]	0.01
PRBC (units)	9 [5, 16]	6 [4,10]	0.001
FFP (units)	2 [0, 6]	3 [0, 6]	NS
Platelet (pools)	2 [1, 3]	1 [0, 2]	0.001
Total transfusion (units)	15 [8, 25]	10 [6, 18]	0.02

*FBNc*: Fibrinogen concentrate; *SOFA*: Sepsis organ failure assessment; *OR*: Operating room; *ICU*: Intensive care unit; *INR*: International normalized ratio; *PRBC*: Packed red blood cell; *FFP*: Fresh frozen plasma; Quantitative variables are expressed as a median [interquartile range]. Qualitative variables are expressed as a number (percentage).

### First primary endpoint: total number of transfused units

Patients from the FBNc group (N = 71) received 2 [2,4] g of FBNc. After administering FBNc, fibrinogen levels increased from 1.3 [0.9, 2.3] g/L to 1.8 [1.2, 2.3] g/L (p = 0.13), and both bleeding (only reliably measured for 33 patients) and blood transfusion volumes were significantly reduced (Table 2).

**Table 2:** **Laboratory values and transfused units before and after FBNc administration**

Variables	Pre-FBNc	Post-FBNc	P value
pH	7.30 [7.28, 7.32]	7.32 [7.26, 7.36]	0.03
Base excess (mEq/L)	-4.3 [-8.0, -3.0]	-4.0 [-7.0, -2.0]	NS
Creatinine (mg/dl)	0.9 [0.7, 1.2]	0.9 [0.7, 1.3]	NS
Platelets (x 10^9^/L)	93 [48, 156]	80 [59, 114]	NS
Haemoglobin (g/L)	85.0 [71.0, 106.0]	90.0 [76.0, 100.0]	NS
Prothrombin time (s)	19.2 [14.0, 31.9]	18.0 [15.4, 24.1]	NS
Thromboplastin time (s)	46.1 [33.7, 98.6]	41.3 [32.6, 58.2]	0.02
Fibrinogen (g/L)	1.3 [0.9, 2.3]	1.8 [1.2, 2.3]	NS
INR	1.7 [1.2, 2.8]	1.6 [1.3, 2.0]	NS
***Outcome variables***			
PRBC (units)	4 [3, 6]	2 [1, 3]	0.00
FFP (units)	1 [0, 2]	0 [0, 2]	NS
Platelet (pools)	1 [0, 2]	0 [0, 1]	0.00
Total (units)	6 [5, 9]	3 [1, 6]	0.00
Bleeding (mL)*	1000 [670, 2000]	450 [242, 700]	0.00

Only patients receiving FBNc (N = 71) are considered (within-group comparison). *Bleeding was measured accurately only in 33 patients. (See caption for Table 1 for details).

Subjects included in FBNc group were compared with those from the comparator group (N = 72). As depicted in Table 1, patients from the FBNc group were significantly sicker (higher SOFA), presented a more altered haemostasis, were more frequently transfused, and had a higher mortality. However, in the multivariate regression analysis, only high SOFA score and low fibrinogen levels upon admission showed an independent association with total number of transfused units (primary endpoint) (Table 3).

**Table 3:** **Multiple linear regression analysis (ANOVA) for variables involved in total transfusion**

Variables	B coefficient	95% CI	p value
SOFA upon admission	0.75	0.08 – 1.43	0.02
Fibrinogen level upon admission	- 2.7	- 4.68 – - 0.78	0.01

*CI*: Confidence interval; *SOFA*: Sepsis organ failure assessment.

As severity of illness (as assessed by SOFA), age and diagnoses could remain as strong confounder variables, data were re-analysed after performing a pair-matching. Seventy-six patients (38 in each group) were successfully matched, and no differences with respect to number of transfused units or mortality between groups were observed (Table 4).

**Table 4:** **Matching, baseline and outcome variables for patients receiving (FBNc group) or not (comparator group) FBNc**

Variables	FBNc N = 38	Control N = 38	P value
Age (years)	62 [55, 68]	62 [57, 73]	NS
SOFA	6 [4, 8]	5.5 [4, 8]	NS
Gender (males)	26 (68.4)	31 (81.5)	NS
Haemorrhage occurring outside the OR or ICU	10 (26.3)	10 (26.3)	NS
**Diagnoses**			
Cardiac surgery	21 (55.3)	21 (55.3)	
Liver transplantation	7 (18.4)	7 (18.4)	
Gastrointestinal bleeding	10 (26.6)	10 (26.6)	NS
***Variables at beginning of bleeding***			
pH	7.30 [7.28, 7.33]	7.31 [7.26, 7.37]	NS
Base excess (mEq/L)	-4.7 [-8.0, -3.0]	-4.0 [-6.0, 0.4]	NS
Creatinine (mg/dL)	0.8 [0.7, 1.3]	0.9 [0.7, 1.4]	NS
Leucocytes (x 10^9^/L)	7.1 [4.7,11.7]	9.7 [7.3, 15.0]	0.04
Platelets (x 10^9^/L)	112 [72, 173]	98 [63, 144]	NS
Haemoglobin (g/L)	84.5 [72.0, 108.0]	70.0 [64.0, 75.0]	0.00
Prothrombin time (s)	18.0 [13.0, 28.0]	18.0 [14.0, 21.0]	NS
Thromboplastin time (s)	43.5 [32.4, 63.8]	47.7 [36.2, 59.2]	NS
Fibrinogen (g/L)	1.7 [0.9, 2.3]	2.0 [1.5, 3.3]	0.04
INR	1.6 [1.2, 2.5]	1.5 [1.2, 1.8]	NS
***Outcome variables***			
In-hospital mortality	13 (34.2)	10 (26.3)	NS
Length of hospital stay (days)*	15 [7, 32]	22 [14, 29]	NS
PRBC (units)	8 [5, 12]	6 [5, 12]	NS
FFP (units)	2 [0, 5]	3 [0, 5]	NS
Platelet (pools)	1 [0, 2]	2 [1, 3]	NS
Total transfusion (units)	11 [6, 20]	10 [6, 16]	NS

Only 76 patients were successfully pair-matched (38 pairs). (See caption for Table 1 for details).

### Second primary endpoint: achievement of fibrinogen levels ≥2 g/L, within a 24-hours period after activating the MTP (N = 143 patients)

Patients who maintained a fibrinogen levels of less than 2 g/l (N = 64), presented a more altered haemostasis, were more frequently transfused, and had a higher mortality (NS) than those who not (N = 77). Fibrinogen levels before of activation of MTP were also significantly lower, and they received higher doses of FBNc (Table 5). After adjusting, only fibrinogen level on admission was directly associated with achieving an optimal fibrinogen level ≥2 g/L (AOR 3.29 [CI 1.95 - 5.56]; p < 0.0001). However, neither the administered doses of FBNc nor the total number of transfused units influenced the achievement of optimal fibrinogen levels.

**Table 5:** **Patients’ characteristics according to the achievement of an optimal fibrinogen level ≥2 g/L (maximum fibrinogen level within 24 hours after the onset of bleeding)**

Variables	Fibrinogen ≥ 2 g/l N = 77	Fibrinogen < 2 g/l N = 64	P value
Age (years)	61 [48, 70]	59 [44, 67]	NS
SOFA	5 [3, 8]	6 [4, 9]	NS
Gender (males)	54 (54.5)	45 (45.5)	NS
FBN administration	0 [0, 2]	2 [0, 3]	0.00
Haemorrhage occurring outside the OR or ICU	22 (28.5)	26 (40.6)	NS
**Diagnoses**			
Cardiac surgery	43 (55.8)	28 (43.8)	
Liver transplantation	12 (15.6)	10 (15.6)	
Gastrointestinal bleeding	13 (16.9)	18 (28.1)	NS
Other	9 (11.7)	8 (12,7)	
***Variables at beginning of the bleeding***			
pH	7.3 [7.3, 7.4]	7.3 [7.2, 7.3]	0.04
Base excess (mEq/L)	-3 [-7, -2]	-6 [-9, -4]	NS
Creatinine (mg/dl)	0.9 [0.7, 1.6]	0.9 [0.7, 1.3]	NS
Leukocytes (x 10^9^/L)	9.9 [7.0, 14.5]	7.9 [4.7, 12.9]	0.02
Platelets (x 10^9^/L)	109 [65, 164]	88 [50, 126]	0.04
Haemoglobin (g/L)	74.0 [66.7, 85.0]	76.0 [65.0, 87.0]	NS
Prothrombin time (s)	16 [14, 21]	19 [16, 27]	0.02
Thromboplastin Time (s)	38 [31, 55]	58 [33, 74]	0.03
Fibrinogen (g/L)	2.1 [1.5, 3.2]	1.3 [1.0, 1.7]	0.00
INR	1.4 [1.2, 1.8]	1.7 [1.3, 2.4]	0.01
***Outcome variables***			
Length hospital stay (days)	18 [10, 32]	18 [8, 26]	NS
PRBC (units)	7 [5, 9]	12 [6, 18]	0.00
FFP (units)	3 [0, 5]	4 [0, 8]	NS
Platelet (pools)	1 [0, 2]	2 [1, 4]	0.00
Total transfusion (units)	10 [6, 16]	16 [7, 30]	0.00
Mortality	22 (28.6)	27 (42.2)	NS

### Secondary endpoints

Factors associated with increased mortality were also investigated by step forward logistic regression analysis (Table 6). SOFA upon admission, low pH and bleeding occurring outside the operating room or ICU were independently associated with increased mortality. No thromboembolic adverse events associated with FBNc administration were observed.

**Table 6:** **Logistic regression analysis for variables involved in hospital mortality**

Variables	Wald	AOR [CI 95%]	p value
SOFA on admission	11.4	1.31 [1.12 - 1.53]	0.01
pH at the onset of bleeding	10.5	0.00 [0.00 - 0.0001]	0.01
Bleeding occurring outside the OR or ICU	6.9	3.73 [1.39 - 9.97]	0.002

*CI*: Confidence interval; *ICU*: Intensive care unit; *OR*: Operating room; *SOFA*: Sepsis organ failure assessment.

## Discussion

Fibrinogen concentrate administration has been found efficacious at reducing transfusion requirements in trauma and surgical bleeding patients, when used as a first line therapy within a goal-directed coagulation management algorithm, based on point-of-care testing [8, 9, 14]. However, less is known regarding the utility of FBNc for controlling on-going haemorrhage and coagulopathy in non-trauma patients in whom a MTP has failed in improving the haemostasis and halting blood loss.

When assessed by adjusted analyses, our data showed that fibrinogen level upon admission was the only variable independently associated with both the global number of transfused units and the achievement of a target fibrinogen level of at least 2 g/L. Low and late dosage of FBNc (roughly 25 mg/kg) was found insufficient for attaining any of these endpoints. In agreement with published evidence on its high safety profile, even if administered at high doses (100 mg/kg) [1, 6, 8], no FBNc-associated thromboembolic event was observed.

As in other non-randomised studies [1, 3], we observed a non-adjusted significant decrement of the total number of transfused units after administering FBNc (N = 71 patients, within-group comparison) (Table 2). However, after adjusting, only the severity of illness, as assessed by SOFA, and low fibrinogen levels on admission, but not FBNc administration, were independently associated with blood component requirements (Table 3). Additionally, no differences in overall transfused units were observed after performing a paired-matched between-group comparison (Table 4).

Data on the use of massive transfusion protocols (MTP), outside of the trauma setting, are scant. The activation of MTP allows a faster and uniform issuing of blood products, though clinical outcome remains poor [16, 17], and early use of FFP to PRBC transfusion ratios of 1:1 or 1:2 ha become widespread [2], though the European guidelines for management of severe perioperative bleeding [10] do not provide precise recommendations either for plasma transfusion or for any specific plasma: RBC transfusion ratio. In contrast, these guidelines definitely recommend the use of predefined algorithms based on POC coagulations monitoring assays to guide haemostatic interventions aimed at improving outcome in elective surgery (1C) [10].

Regarding FBNc, with the exception of US guidelines, published guidelines suggest or recommend its administration in bleeding patients with either fibrinogen levels below 1.5 - 2.0 g/L or FIBTEM ROTEM evidence of functional fibrinogen deficiency [10–13, 18–20], despite this is not an approved indication for FBNc in all countries [14]. Therefore, the risk to benefit balance of using FBNc as part of the MTP should be discussed at any institution.

We observed that both low fibrinogen levels on admission and maximum fibrinogen level within a 24-hours period after MTP activation were inversely and independently correlated with the number of transfused units (Figure 3): blood component transfusion increased by almost 3 units per each g/L decrease in admission fibrinogen level (Table 3). This is in agreement with the significant though weak-to-moderate correlation (R = -0.40) between pre- and postoperative fibrinogen levels and postoperative blood loss in cardiac surgery, found in a recent meta-analysis [21]. However, FBNc administration did not reduce the use the allogeneic blood products in our patients (Table 3). More important, administration of low doses of FBNc was not associated with reaching an optimal fibrinogen level at least of 2 g/L. Several factors may have accounted for the apparent lack of efficacy of FBNc in this scenario.

First, these were bleeding patients (roughly one half undergoing cardiovascular or hepatic surgery) who had already received at least one massive transfusion package before administering FNBc. Our patient management protocol is opposed to that for patients included into a goal-directed therapy algorithm. For the later, two recent meta-analysis of 6 and 12 RCTs, respectively, demonstrated that administration of variable, goal-directed doses of FBNc effectively decreased transfusion requirements (**6,15**).

Second, the administered FBNc doses were at the lower range of guidelines’ recommendations [10]. We administered 2 [1,6] g of FBNc reaching a post infusion fibrinogen level of 1.8 g/L. However, in 25% of patients, fibrinogen remained lower than 1.2 g/L within 12-hours following administration (Table 2). Moreover, for the whole sample (N = 141), only 53.8 % attained fibrinogen levels ≥2 g/L, despite massive transfusion, with or without the additional FBNc.

Higher doses (above 50 mg/kg) have been shown to reduce bleeding [6, 8, 9] and improve coagulopathy [22, 23]. Therefore, it is possible that FBNc doses were too low, resulting in inappropriate fibrinogen levels for improving haemostasis.

Third, independently of the threshold and target levels for FBNc administration, timing is also an important issue, which is not accounted for in current guidelines. In patients with massive haemorrhage, waiting for standard laboratory fibrinogen assessment invariably resulted in late FBNc administration [24]. Even if FIBTEM ROTEM is used, a minimum running time will be needed (10–15 minutes), albeit significantly shorter than that for conventional laboratory [24].

We administered FBNc belatedly, when patients had already received 6 [5,9] units of blood components and had severe coagulopathy. Although speculative, it is conceivable that FBNc efficacy was sub-optimal because of late coagulopathy affecting platelets, proenzymes, and the fibrinolytic system [25]. Earlier administration of coagulation factor concentrates might have resulted in improved treatment of coagulopathy and avoided the side effects of plasma administration [24].

Lastly, a selection bias could have contributed to the apparent lack of efficacy of FBNc administration observed in our series. As depicted in Figure 1, clinicians were free for administering plasma, FBNc or both. In fact, FBNc was prescribed after transfusion of blood components had failed to correct coagulopathy and bleeding. Therefore, FBNc administration might actually be a surrogate marker of severity of illness.

Some limitations of our study should be acknowledged. First, its retrospective, uncontrolled nature does not allow an adequate estimation of the impact of FBNc therapy on transfusion requirements. However, adjusted analyses and matched comparison tried to overcome this bias. Second, blood transfusion is a surrogate marker of blood loss, and therefore changes in blood losses should have reflected better the efficacy of FBNc. Unfortunately, it was difficult to accurately measure the amount of blood loss. Third, patients who died within 24-hours from the onset of massive bleeding were excluded from the study. Lastly, we reviewed the FBNc efficacy at treating massive haemorrhage in non-trauma patients with heterogeneous diagnoses.

Despite the abovementioned limitations, our study has also important strengths. This is one of the few studies dealing with the use of FBNc, as a haemostatic intervention, in patients with severe perioperative bleeding managed with a MTP, and reporting on a relatively large sample of patients. Moreover, unlike most published studies, we performed multivariate analyses and used a comparator group to document FBNc efficacy.

## Conclusion

Our results suggest that the late administration of low doses of FBNc is not useful in massively transfused patients with severe coagulopathy. However, independent associations between severity of illness and low levels of fibrinogen upon admission with transfusion requirements were found. Therefore, it is conceivable that earlier administration of higher doses, based on a goal-directed, POC-guided algorithm, would improve FBNc effectiveness in this clinical setting. We recognize that our results neither suggest nor support the inefficacy of FBNc, but rather the probable inefficacy of its inappropriate use. Prospective studies on the role of FBNc administration to non-trauma patients with severe bleeding are urgently needed.

### Key messages

– The late administration of low doses of fibrinogen concentrate is not useful in massively transfused patients with severe coagulopathy.
– Low fibrinogen upon admission and maximum fibrinogen level were inversely associated with transfusion rate. Requirements of blood component transfusion increased by 3 units per each g/L decrease in admission fibrinogen levels.
– Our data also suggest that both earlier administration and higher doses could improve FBNc effectiveness in massively transfused patients with coagulopathy.

## Abbreviations

aPTT: Activated thromboplastin time
FBNc: Fibrinogen concentrate
FIBTEM-ROTEM: Functional fibrinogen assessment by rotational thrombelastometry
FFP: Fresh frozen plasma
INR: International normalized ratio
MTP: Massive transfusion protocol
PBRC: Packed red blood cell
PLT: Platelets
POC: Point-of-care
RCTs: Randomised controlled trials
SOFA: Sepsis organ failure assessment
TEG: Thromboelastrography
ROTEM: Rotational thrombelastometry.
